# β-Bursting as a Sensitive Neural Marker of Inhibitory Control in Healthy Older Adults: A Linear Mixed-Effects Modeling and Threshold-Free Cluster Approach

**DOI:** 10.1523/JNEUROSCI.1151-25.2026

**Published:** 2026-02-24

**Authors:** Aliya C. M. Warden, Damian Cruse, Craig J. McAllister, Hayley J. MacDonald

**Affiliations:** ^1^School of Sport, Exercise and Rehabilitation Sciences, College of Life and Environmental Sciences, University of Birmingham, Birmingham B15 2TT, United Kingdom; ^2^Centre for Human Brain Health, University of Birmingham, Birmingham B15 2TT, United Kingdom; ^3^School of Psychology, College of Life and Environmental Sciences, University of Birmingham, Birmingham B15 2TT, United Kingdom; ^4^Department of Biological and Medical Psychology, University of Bergen, Bergen 5009, Norway

**Keywords:** β-bursts, β-oscillations, electroencephalography, impulse control, response inhibition

## Abstract

Inhibitory control is essential for adaptive behavior and declines with age, yet the underlying neural dynamics remain poorly understood. The β-rhythm (15–29 Hz) is associated with inhibitory signaling within the frontobasal ganglia network. Recent evidence suggests that transient β-bursts support inhibitory performance but are often masked by conventional trial-averaged β-power analyses. A recently developed analysis approach, combining linear mixed-effects modeling and threshold-free cluster enhancement (LMM–TFCE), was applied to examine trial-by-trial β-bursting activity associated with response inhibition and initiation in older adults. Twenty healthy older adults (nine females) performed a bimanual anticipatory response inhibition task, while electroencephalography and electromyography were recorded to capture β-activity (β-burst rate/volume; averaged β-power) and muscle bursting dynamics, respectively. Our analysis revealed distinct β-bursting signatures absent in averaged β-power data. During bimanual response inhibition, parieto-occipital β-bursting preceded bilateral frontocentral β-bursting, consistent with initial attentional processes prior to broader inhibitory network engagement. Moreover, a link was established between right sensorimotor β-bursting and muscle bursts during stopping, indicating rapid cortical suppression of initiated motor output. β-burst volume proved uniquely sensitive to response withholding, with early left frontal activity supporting preparatory suppression mechanisms. A further link between increased parieto-occipital β-burst volume and muscle bursts aligned with top–down inhibitory signaling to support visuomotor stabilization and prevent premature response release. These results underscore the sensitivity of β-bursting to both the timing and context of inhibitory demands in healthy aging. Future research will help establish the potential of β-bursting, combined with LMM–TFCE analysis, as a clinically relevant marker of impulse control dysfunction.

## Significance Statement

Our novel application of an advanced statistical framework revealed distinct spatiotemporal β-bursting patterns during response inhibition and response withholding in healthy older adults, which were not captured by averaged β-power. Identifying a further link between cortical β-bursting and muscle-level suppression, the findings offer a mechanistic account of how the brain halts action in real time in older adults. This work provides a sensitive, trial-level framework for studying β-burst measures in general, as well as inhibitory control across aging and clinical populations.

## Introduction

Suppressing inappropriate actions is fundamental to goal-directed behavior and is commonly assessed through response inhibition paradigms (Verbruggen et al., 2019). Classic Go/No-Go and Stop-Signal tasks examine the withholding or cancelation of externally cued responses. Performance on these tasks recruit a right-lateralized frontobasal ganglia network (Aron et al., 2014, 2016) and reveal age-related slowing of inhibitory control (Rey-Mermet and Gade, 2018). However, these paradigms cannot capture the internally timed nature of everyday actions. In the increasingly popular anticipatory response inhibition task (ARIT; He et al., 2022), response withholding is proactively maintained and then strategically released for response initiation synchronized with a stationary target on Go trials, while Stop trials trigger reactive inhibition of the anticipated response via a stop-signal. Directly contrasting Go and Stop trials differentiates proactive versus reactive inhibitory control (Meyer and Bucci, 2016).

β-oscillations, a hallmark of motor-related neural activity, are linked with GABAergic inhibition (Groth et al., 2021). Sensorimotor β-power decreases during movement (movement-related desynchronization) and transiently increases afterward (postmovement rebound; Kilavik et al., 2013). However, growing evidence suggests raw β-band activity is better characterized by transient, burst-like events (<150 ms) than overall power (Sherman et al., 2016; Shin et al., 2017; Barone and Rossiter, 2021). Spatiotemporal patterns of β-bursting may support cognitive control through transient, functional inhibition (Zich et al., 2023; Lundqvist et al., 2024). In young adults, sensorimotor β-bursting is thought to reflect an inhibited motor state during response withholding, which is released to initiate movement, but can be rapidly reinstated to stop movement following frontal control signals. Increased frontocentral β-burst rate (Wessel, 2020) and volume (incorporating burst duration, frequency span, and amplitude; Enz et al., 2021) predict successful stopping, while earlier β-bursts are associated with faster stopping (Hannah et al., 2020). Collectively, frontocentral and sensorimotor β-bursting patterns offer valuable insights into neural–behavioral associations underlying inhibitory control.

Muscle-level measures can capture the downstream effects of these β-bursting neural dynamics. During the ARIT, healthy older adults display premature muscle bursts when withholding the planned response in ∼17% of Go trials and partial bursts on ∼50% of successful Stop trials, reflecting initiation and rapid cancelation of a response (Warden et al., 2025). Partial burst latency provides a single-trial index of stopping (CancelTime; Raud et al., 2022). Jana et al. (2020) reported that right frontal β-bursting precedes CancelTime by ∼37–54 ms, with earlier β-bursts associated with faster CancelTime, linking frontal β-bursting to motor suppression. However, the mechanistic relationship between β-bursts across the wider inhibitory control network and trial-by-trial muscle responses remains unclear.

Current analyses of β-bursting dynamics often rely on subject-wise averages of β-activity that may obscure meaningful trial-level and interindividual differences—particularly in older populations with heterogeneous neural responses (Riccardi et al., 2025). The present study therefore adopted a recently developed analysis framework introduced by Visalli et al. (2024), combining linear mixed-effects modeling (LMM) and threshold-free cluster enhancement (TFCE). By modeling β-features at the single-trial level, LMMs account for within- and between-participant variability, more reliably isolating the effects of interest. Complementing this, TFCE enhances sensitivity to broad yet comparatively subtle clusters of β-activity by weighting both their strength and spatial extent rather than relying on arbitrary thresholds (Smith and Nichols, 2009). Together, these methods offer a more statistically powerful approach for characterizing trial-by-trial β-burst dynamics, especially across heterogeneous responses.

The first aim of this study was to apply a sensitive statistical framework combining LMM and TFCE (LMM–TFCE) techniques to contrast β-burst activity during response inhibition versus initiation in healthy older adults performing the ARIT. Given their sensitivity to inhibitory control processes, β-burst rate and volume were quantified and compared with conventional averaged, normalized β-power. We hypothesized that increases in right frontocentral β-bursting would precede subsequent bilateral sensorimotor β-bursting during response inhibition. To investigate downstream effects of β-bursting, the second aim applied this framework to characterize the potential functional link between cortical β-bursting and muscle bursting during response inhibition and withholding.

## Materials and Methods

### Participants

Twenty healthy older adults (69.3 ± 5.8 years, nine females, 18 self-reported as right-handed) were recruited as part of a larger preregistered study (https://doi.org/10.17605/OSF.IO/KC2H3). While the larger study includes comparisons between people with Parkinson's disease and healthy older adults, this paper reports the novel application of LMM–TFCE analyses in healthy older adults only.

Participants were recruited via the Birmingham 1000 Elders group and local community hubs. Participants were recruited if aged 40–80, had no history of neurological conditions, had no reported vision impairment that was not corrected (e.g., with glasses), and scored >23 on the Montreal Cognitive Assessment (*M* = 27.4 ± 1.6; Karlawish et al., 2013).

All participants gave written informed consent and received monetary compensation for their participation. The study received favorable opinion from an NHS Research Ethics Committee (South-East Scotland REC 02, IRAS ID: 328075) and was conducted in accordance with the Declaration of Helsinki.

### Behavioral task

**Figure 1.**
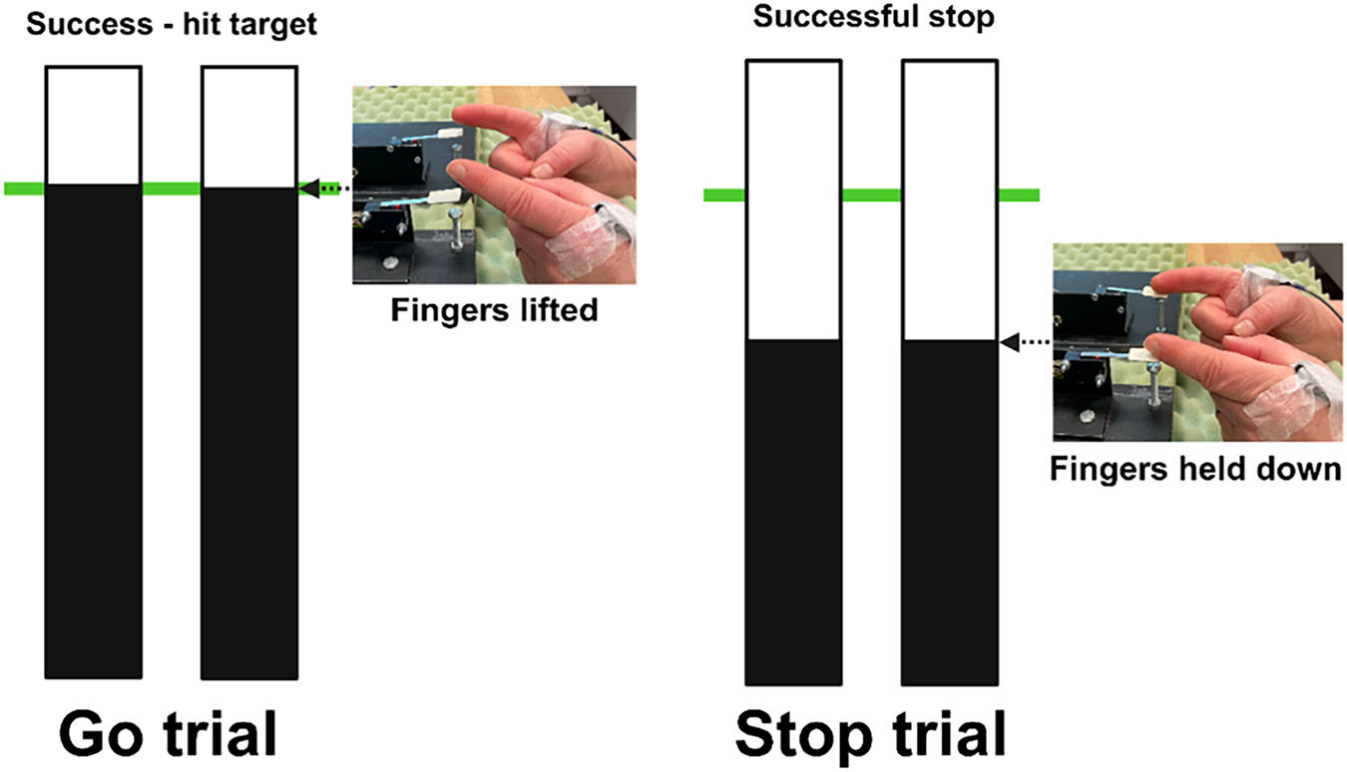
The ARIT. During Go trials, participants initially held their index fingers on the respective switches and were instructed to lift their fingers once rising indicators reached the target line (at 800 ms). During Stop trials, the indicators stopped prematurely before the target and participants were instructed to inhibit the prepared finger lifts.

As described previously (Warden et al., 2025), the current study employed a bimanual version of the ARIT (Fig. 1), programmed in MATLAB (R2019b, The MathWorks) and operated via custom microswitches. Participants were seated ∼0.6 m from a monitor displaying two vertical indicators, resting their forearms on a table midway between supination and pronation. The medial aspect of each index finger was used to lightly depress the two switches, with the left and right indicators corresponding to the respective fingers. After a 2 s delay, both indicators started to move upward from the bottom at equal rates.

The task was primarily composed of Go trials (67%, 320 trials), where participants released both switches to intercept the rising indicators with a stationary target line at 800 ms (Fig. 1, left). Go trials were classed as “on-target” if both indicators were within ±50 ms of the target line. During Stop trials (33%, 160 trials), both indicators stopped before reaching the target (Fig. 1, right), cueing participants to inhibit the prepared bimanual movement. Following established practice (He et al., 2022), Stop trials employed an adaptive staircase algorithm that dynamically adjusted the stop-signal delay (SSD) in response to trial-by-trial performance, ensuring consistent task difficulty across participants. The 2:1 trial ratio helped ensure a prepotent Go response. Immediate visual feedback reinforced performance, and participants were instructed to respond promptly and accurately to prevent strategic slowing while maintaining relaxed muscles until response initiation to reduce baseline activity.

### Recording and preprocessing

#### Electroencephalography

Continuous scalp electroencephalography (EEG) was recorded with a 128-channel Biosemi ActiveTwo system (Biosemi, Amsterdam, The Netherlands). Two additional reference electrodes were recorded from the mastoid processes. EEG data were digitized at a sampling rate of 1,024 Hz and preprocessed using a combination of custom MATLAB scripts (R2019b, The MathWorks), the EEGLAB toolbox (Delorme and Makeig, 2004; http://sccn.ucsd.edu/eeglab), and the Fully Automated Statistical Thresholding for EEG artifact Rejection procedure (FASTER; Nolan et al., 2010; http://sourceforge.net/projects/faster). The data were first digitally filtered between 0.5 and 40 Hz, referenced to the average of the mastoids, and segmented into epochs from 500 ms before the indicators started to rise until the end of the trial (+1,000 ms).

Artifact rejection was performed using automated procedures based on FASTER (Nolan et al., 2010). First, channels with absolute *z*-scores >2.5 on any of the following measures were removed: voltage variance, mean correlation with other channels, and Hurst exponent. Second, trials containing nonstationary artifacts were excluded using the same threshold, based on mean voltage range across channels, mean voltage variance, and deviation of trial average voltage from the grand average. Third, independent component analysis was conducted on the remaining data (via the EEGLAB runica algorithm), and the ADJUST toolbox (Mognon et al., 2011) was used to automatically identify and remove components with the expected spatial and temporal features of blinks, eye movements, and generic discontinuities. Upon visual inspection, any trials with artifacts that were not effectively cleaned by the above procedure were discarded. Fourth, any previously removed channels were interpolated back into the data. Finally, data were rereferenced to the average of all channels. After data exclusions, a median of 159 on-target Go trials (range, 74–245) and 80 successful Stop trials (range, 65–83) were included in the analysis. There was no apparent effect of age on data quality (*r* = −0.226; *p* = 0.339).

To enhance spatial resolution for β-burst detection, EEG data were transformed using the current source density method (Kayser and Tenke, 2006; https://psychophysiology.cpmc.columbia.edu/software/CSDtoolbox/index.html).

#### Electromyography

The lift response required participants to abduct their index fingers using the first-dorsal interosseous (FDI) muscle as the agonist. Surface electromyography (EMG) was recorded via the Biosemi ActiveTwo amplifier (Biosemi) using two flat-type active electrodes placed over each FDI muscle, sampled at 1,024 Hz. The scalp reference electrodes (CMS/DRL) were used as the ground. The difference between the two electrode signals was amplified, bandpass filtered (50–450 Hz), and processed with custom MATLAB scripts and EEGLAB. EMG data were segmented into 1 s epochs from when the indicators started to rise until the end of each trial.

### Experimental design and statistical analysis

#### Task performance

Go trials without a lift response were excluded from analyses. Lift-times were recorded for each index finger via registration of the lifted switches. For each side, mean lift-time was calculated after trimming outliers (±3 SD) (MacDonald et al., 2014). Stop-signal reaction time (SSRT) was calculated using the integration method, estimating the time needed to inhibit the response on Stop trials (Verbruggen et al., 2019). Trimmed lift-times averaged across side for Go trials were rank ordered and the *n*th lift-time selected, with *n* obtained by multiplying the number of lift-times by the probability of a response on a Stop trial. The time at which the staircase procedure stopped the indicators to achieve 50% success (*staircased* SSD) was subtracted from the *n*th lift-time.

#### β-Features

*Time–frequency decomposition*. Time–frequency decomposition was performed using the established methods of Enz et al. (2021) and Wessel (2020). Power estimates were computed across 15 linearly spaced frequencies from 15 to 29 Hz, using complex Morlet wavelets with 4–10 cycles logarithmically spaced. The squared magnitude of the convolved signal was used to obtain power for each electrode and epoch.

*Average β-power*. For wider comparison to earlier studies on β-oscillations during response inhibition, average β-power was calculated. Baseline activity was calculated as average β-power while the switches were pressed down, 100–400 ms before the indicators started to rise. Power estimates were then converted to decibels (dB) relative to this baseline period (baseline normalized).

*β-burst detection*. Transient β-bursts were identified from the raw (nonbaseline normalized) power estimates via an established thresholding method (Hannah et al., 2020; Wessel, 2020; Enz et al., 2021). Peaks of β-activity exceeding two times the median β-power of each trial were detected as β-bursts, posited by Enz et al. (2021) as a sensitive threshold for investigation of the stopping process. This approach allows for a different threshold per channel, per trial, and per participant, accounting for differences in underlying β-activity between and within individuals.

*Spatiotemporal analysis*. For successful Stop trials, an individualized time window (0–312.5 ms prior to SSRT) was extracted based on each participant's SSRT. Trials with an SSD exceeding 700 ms were excluded to encompass the full range of SSRT values. This individualized time-locking aligns β-features more consistently across the study group, as they are temporally anchored to each participant's stop-signal processing dynamics. For on-target Go trials, a window of equal length was taken relative to the earliest lift-time on each trial, capturing β-activity as each participant withholds and then initiates the response.

To assess changes in β-activity during the response withholding and inhibition phases, 10 equal time bins of 31.25 ms length were defined (i.e., 32 samples at 1,024 Hz), with 0 ms representing the SSRT/lift-time. β-power was computed over whole epochs before being subdivided into time bins. Two β-burst features were extracted in each time bin: (1) β-burst rate, the sum of the number of β-bursts at each electrode, and (2) β-burst volume, the area under the curve of suprathreshold datapoints, individually calculated for each frequency and subsequently summed up over all frequencies (Enz et al., 2021). While β-burst volume captures burst duration, frequency span, and amplitude as a composite measure, separate metrics for β-burst amplitude and duration were also extracted (Figs. S1, S2).

**Figure 2.**
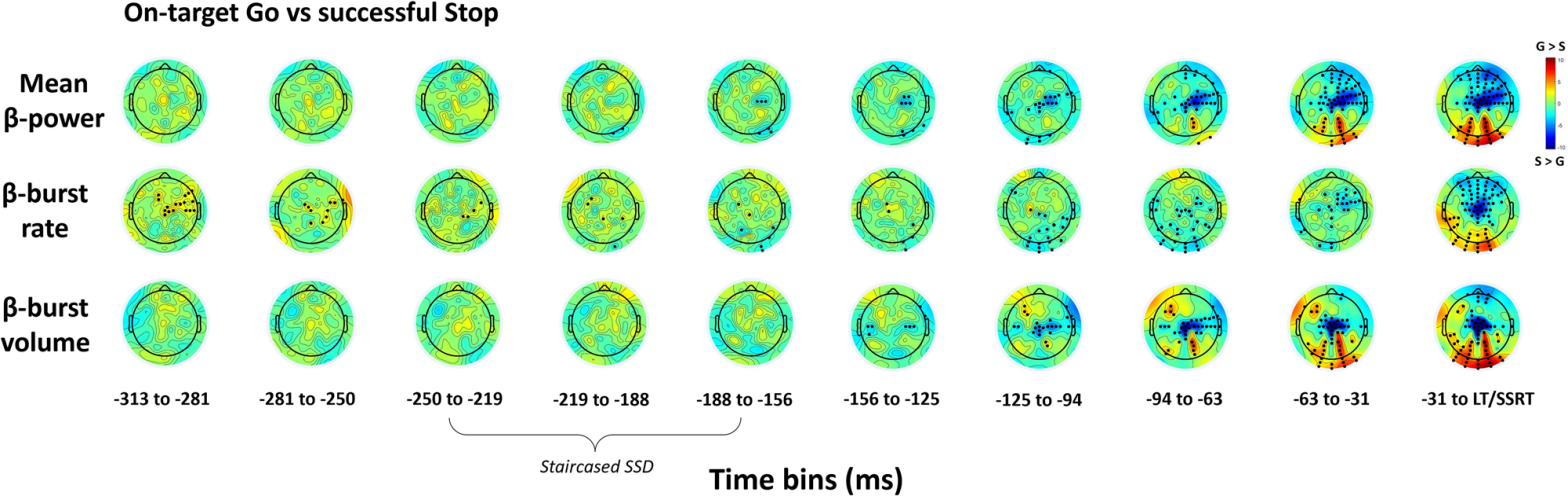
Topographical comparison of β-features between Go and Stop trials. Topoplots of *T* statistics for average β-power, β-burst rate, and β-burst volume across 10 ∼31 ms time bins leading up to the lift-time (LT) or SSRT in on-target Go trials versus successful Stop trials. The β-band frequency range was 15–29 Hz, and β-bursts were detected using a 2 × median power threshold. As per the color bar, red indicates a relative increase in β-activity in Go trials (G) while blue indicates a relative increase in Stop trials (S). Black dots refer to significant electrodes following TFCE permutation testing (*p* < 0.05). SSRTs ranged from 180 to 239 ms, with staircased SSD values falling across several timepoints, as indicated.

To visualize the topographical distribution of β-activity over the course of the trial, β-power, β-burst rate, and β-burst volume were plotted across time bins for on-target Go versus successful Stop trials in a topographical grid representing the scalp surface (Fig. 2).

#### Muscle bursting

Following data collection, three participants were excluded from the muscle bursting analyses due to low signal-to-noise ratio in the EMG data (*n* = 17). EMG data processing was performed using MATLAB (2022a, The MathWorks). Trials with baseline root-mean-squared (rms) EMG exceeding 30 µV between 300 and 400 ms after the indicators started to rise were excluded from the analysis. As per the second study aim, ineffective bursts of muscle activity (i.e., those that did not trigger a lift response) were identified on a trial-by-trial basis across Go and successful Stop trials.

Muscle bursts were defined as instances when the smoothed rectified EMG amplitude surpassed 15 SD of the baseline (rmsEMG at 0–400 ms) and remained above threshold for ≥5 ms. On Go trials, the main burst generating the lift response was identified as the last burst with an onset occurring before the recorded switch release. Accordingly, “premature bursts” were identified as suprathreshold EMG activity prior to the main lift response. On successful Stop trials, “partial bursts” represented muscle activity which decreased in amplitude before generating sufficient force to trigger the lift response. Bursts occurring within an individualized time window (±3 SD of trimmed Go lift-times) were classed as partial bursts, indicating preparation to respond. CancelTime was calculated as the difference in milliseconds between the SSD and peak amplitude of the partial burst (e.g., when muscle activation begins to decrease; Raud et al., 2022).

The precise onset and offset times of these muscle bursts were automatically detected using a single-threshold algorithm (Hodges and Bui, 1996). Bursts separated by <15 ms were merged into a single burst. Detected bursts were manually reviewed on a trial-by-trial basis. Burst trial incidence was calculated as the percentage of traces with ≥1 burst.

#### LMM

To examine β-activity during response inhibition versus initiation (Aim 1), LMMs were developed using the lmeEEG toolbox (Visalli et al., 2024) in MATLAB (2024b, The MathWorks) for each β-feature (β-power, β-burst rate, β-burst volume). Briefly, this method fits a LMM to each electrode-by-timepoint, modeling, and removing random effects (intercepts only), before conducting mass linear regressions on the marginal data. This approach allows for control of multiple comparisons by permutation testing without the enormous computational costs of fitting large numbers of LMMs. For each LMM, the participant was included as a random effect and trial type (on-target Go vs successful Stop) was included as a fixed effect, with effects coding used for each contrast:

$$\mathrm{EE}G_{\mathrm{ch},t}\;∼\;1\;+\mathrm{TrialType}\;+(1|\mathrm{Participant}).$$

This statistical approach was utilized to effectively address both within- and between-participant variance, modeling β-activity on an individual-trial basis, thereby accounting for its inherent variability. Controlling for random effects in this manner more accurately models the clustered system (Visalli et al., 2024), helping to isolate distinct β-activity patterns during response inhibition versus initiation processes.

To explore potential associations between β-bursting and muscle bursting during response inhibition and withholding (Aim 2), two LMMs were developed on β-bursting dynamics (rate/volume) during trials which did or did not feature muscle bursting. Specifically, for Go and Stop trials separately and for β-burst rate and β-burst volume separately (as extracted above), we included the presence/absence of a muscle burst as a fixed effect and participant as a random effect as follows:

$$\mathrm{EE}G_{\mathrm{ch},t}\;∼\;1\;+\mathrm{MuscleBurst}\;+(1|\mathrm{Participant}).$$

#### TFCE

Following LMM, TFCE (Mensen and Khatami, 2013) permutation testing was performed to control for multiple comparisons and identify significant (*p* < 0.05) clusters within the data. TFCE applies a weighted average between the cluster extent (i.e., number of connected samples) and cluster height (indicative of the magnitude of the statistical effect). This approach enhances sensitivity to broad yet comparatively subtle clusters, which may be missed in conventional cluster permutation tests. The resulting TFCE values are scaled according to the membership status of individual data points within identified clusters, thereby accounting for spatial extent.

#### Code accessibility

The custom MATLAB scripts used in the current study are available on the Open Science Framework: https://osf.io/4yu9n/.

## Results

### Behavioral data

During Go trials, mean lift-times for the dominant (835.7 ± 14.2 ms) and nondominant (831.6 ± 15.6 ms) hands occurred shortly after the target line. On Stop trials, stopping success rates of 51.2 ± 2.4% indicate that the SSD staircasing procedure was effective. Mean SSRT was 210.5 ± 13.9 ms, reflecting response inhibition performance. Behavioral performance on Go and Stop trials was therefore as expected for healthy older adults and consistent with previous studies using the ARIT (Macdonald et al., 2012; Hall et al., 2022).

### β-Features

#### β-Power

**Figure 3.**
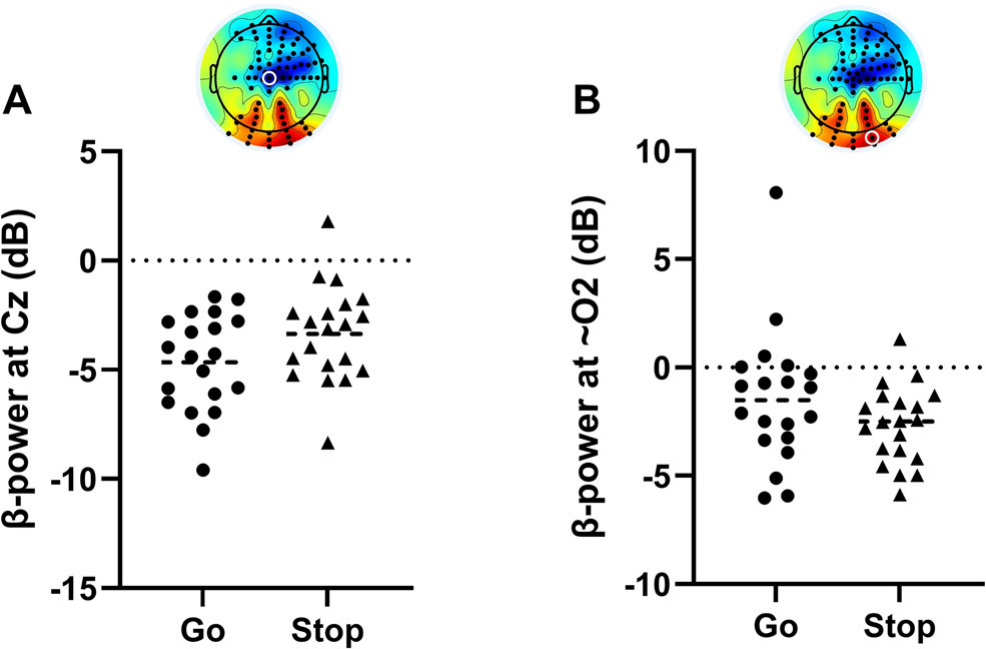
Individual-electrode β-power before response initiation and inhibition. In the final time bin (31 ms to lift-time/SSRT), panel ***A*** shows β-power at a central electrode (Cz [A1]), demonstrating reduced β-desynchronization in successful Stops compared with Go trials. Conversely, panel ***B*** demonstrates relatively increased β-desynchronization over a posterior electrode (close to O2 [A27]) as participants inhibit the response in Stop trials.

The LMM revealed a relative increase in average β-power primarily over central and right frontal electrodes during successful Stop trials compared with on-target Go trials (*p* < 0.05, TFCE-corrected; Fig. 2). At the individual electrode level, this effect reflected reduced β-desynchronization during successful Stop trials, compared with the larger β-desynchronization observed in Go trials as participants prepared to move (Fig. 3*A*). This relative enhancement of right frontal β-power emerged ∼156–188 ms before the SSRT, consistent with engagement of the right-lateralized inhibitory control network during successful stopping.

In contrast, a relative decrease in average β-power over posterior electrodes originated in the right parietal region (62–93 ms before the SSRT) and extended to bilateral parietal and occipital sites as the SSRT approached in successful Stop trials, perhaps reflecting suppression of background sensory noise to focus inhibitory processes.

#### β-Bursting

**Figure 4.**
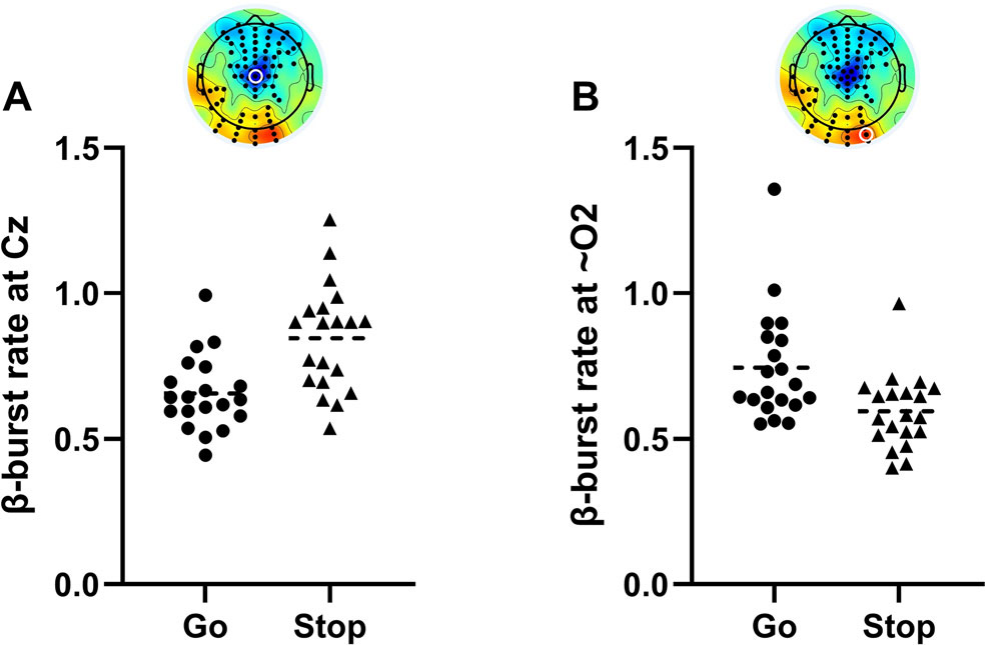
Individual-electrode β-burst rate before response initiation and inhibition. In the final time bin (31 ms to lift-time/SSRT), panel ***A*** shows the β-burst rate at a central electrode (Cz [A1]), demonstrating the relatively increased β-burst rate in successful Stops compared with Go trials. Conversely, panel ***B*** shows a relative decrease in the β-burst rate at a posterior electrode (close to O2 [A27]) as participants inhibit the response in Stop trials.

In support of our hypothesis, inhibition of the prepared response in Stop trials was characterized by a relative increase in frontocentral β-bursting (*p* < 0.05, TFCE-corrected; Fig. 4*A*), whereas β-bursting decreased over posterior areas (Fig. 4*B*). To further confirm that participant-specific spectral properties did not influence the burst detection algorithm, we examined the relationship between spectral slope/offset and β-burst rate and found no significant associations [all *p* > 0.24; calculated from 2 to 40 Hz with FOOOF (Donoghue et al., 2020) via the Fieldtrip toolbox (Oostenveld et al., 2011), averaged across channels]. Interestingly, β-burst dynamics showed distinct spatial and temporal patterns to average β-power.

First, β-bursting presented more bilaterally. Following presentation of the stop-signal, β-burst volume increased across bilateral motor sites (125–156 ms prior to SSRT; Fig. 2). As the SSRT approached, β-bursting became more apparent over right motor areas but ultimately converged on a central motor hotspot, with activity extended to bilateral frontal electrodes. Increases in β-burst volume were particularly focal over a central hotspot and a small cluster of frontal electrodes, perhaps demonstrating greater specificity. In contrast, increases in average β-power remained diffusely distributed across right frontal electrodes.

Second, during successful response inhibition, early-occurring increases in β-burst rate were apparent at bilateral parieto-occipital electrodes (31–125 ms prior to SSRT; Fig. 2), suggesting increased transient β-activity in regions associated with attentional engagement. Interestingly, this pattern was not reflected in average β-power or β-burst volume, which instead demonstrated reduced activation of these sites as the SSRT approached.

There were also differences in β-burst activity during the response withholding phase. In early time bins, there was a relative increase in right frontocentral and sensorimotor β-burst rate in Go trials (absent in average β-power and β-burst volume) which subsequently decreased, perhaps reflecting early preparatory suppression processes. In β-burst volume, however, response withholding was marked by a left frontal hotspot emerging ∼125 ms before the lift, which subsequently decreased as the lift-time approached, perhaps reflecting the later engagement of inhibitory networks to prevent premature response initiation.

### Muscle bursting

**Figure 5.**
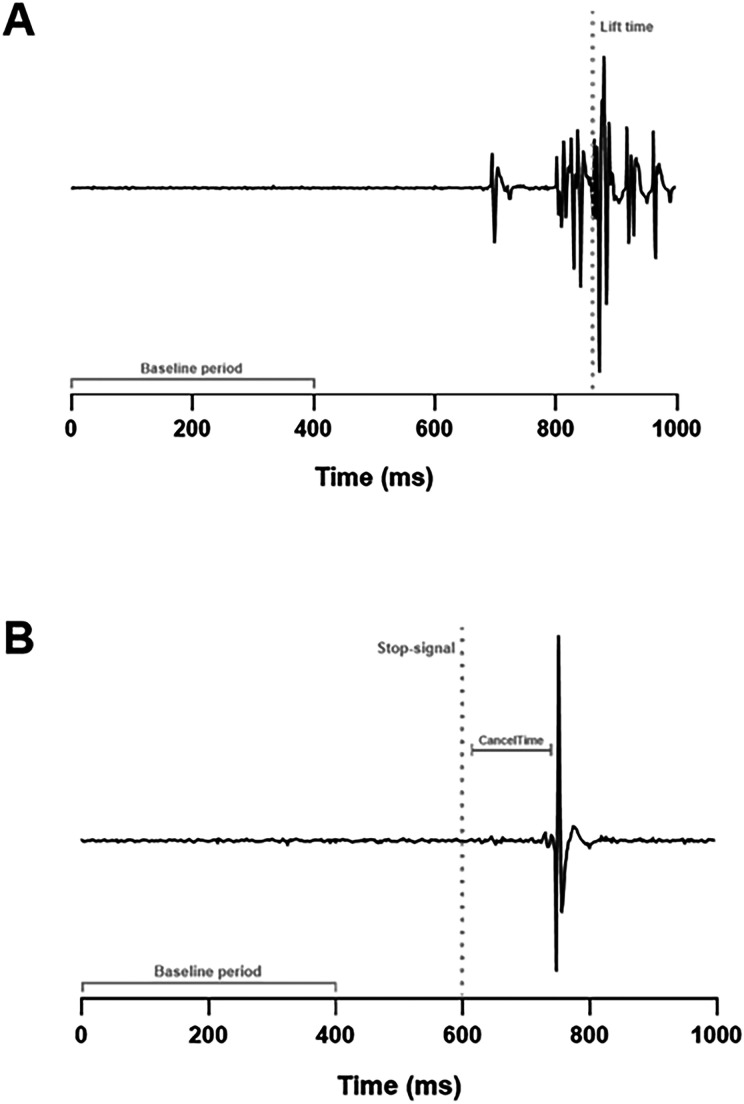
Representative EMG traces of muscle bursting. Panel ***A*** demonstrates a premature muscle burst (∼700 ms) prior to the main lift response (dotted line indicates lift-time). Panel ***B*** demonstrates a partial muscle burst following the stop-signal (dotted line) around when the response would have occurred (∼750–800 ms).

As expected, healthy older adults demonstrated both premature bursts prior to the lift response on Go trials (Fig. 5*A*) and partial bursts when the lift would have occurred on successful Stop trials (Fig. 5*B*). During response withholding, 18.4 ± 10.0% of Go trials contained at least one premature burst, occurring 67–181 ms before the target on average (i.e., ∼100–210 ms before the lift-time). During response inhibition, 51.5 ± 20.4% of successful Stop trials presented with at least one partial burst. Mean CancelTime was 135.2 ± 16.6 ms (∼75 ms before SSRT), and, as expected, there was a positive correlation between CancelTime and SSRT (*r* = 0.578; *p* < 0.001).

### Single-trial integration of β- and muscle bursts

**Figure 6.**
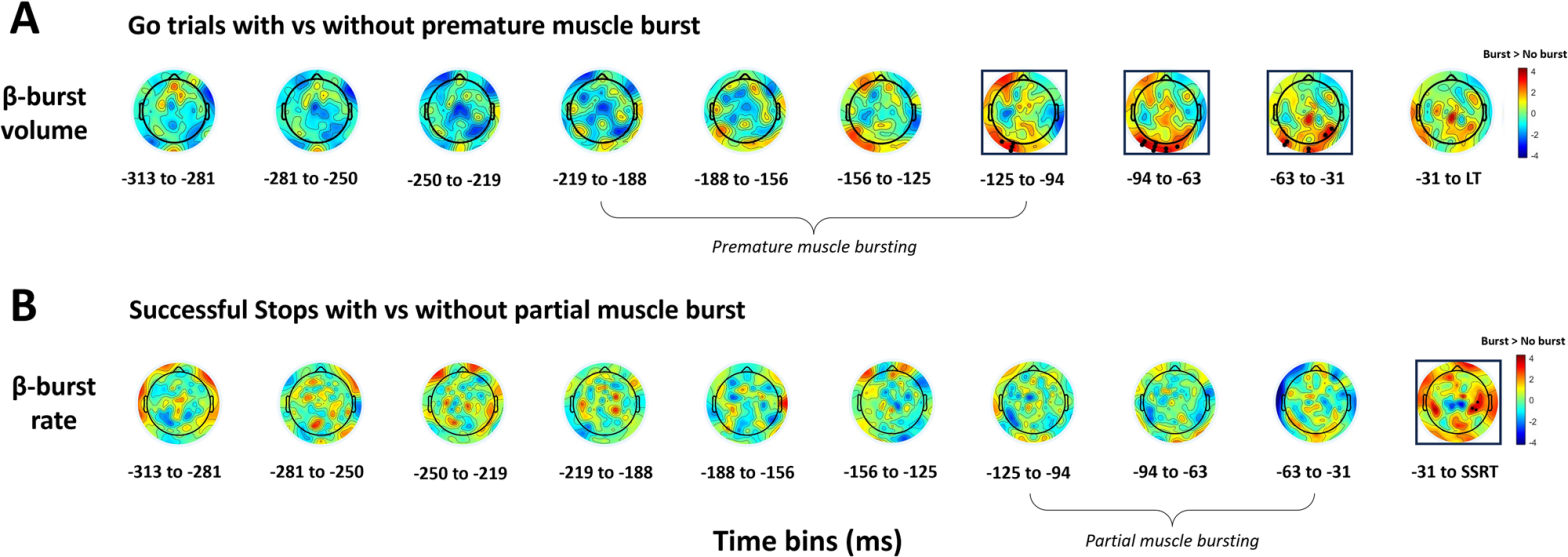
Topographical comparison of β-features in trials with versus without muscle bursts. *T* statistics are shown for β-burst volume and rate across 10 ∼31 ms time bins preceding the lift-time (LT) on Go trials (***A***) and SSRT on successful Stop trials (***B***). Panel ***A*** shows relative increases in parieto-occipital β-burst volume in Go trials with premature muscle bursts versus trials without bursting (significant plots outlined in black). Panel ***B*** shows relative increases in the right motor β-burst rate in successful Stop trials with partial muscle bursts. Black dots refer to significant electrodes following TFCE permutation testing (*p* < 0.05). The average timing of premature and partial muscle bursts is indicated with brackets.

During response withholding, the LMM revealed a significant increase in β-burst volume across (left) parieto-occipital electrodes in Go trials with premature muscle bursting compared to those without (*p* < 0.05, TFCE-corrected; Fig. 6). This effect occurred after the typical presentation of premature muscle bursts, suggesting a reactive mechanism in response to this erroneous motor output. During response inhibition, the LMM revealed a significant increase in the β-burst rate (but not volume) in a small cluster of electrodes (close to C4/CP4 [B17–18, B23]) over right motor areas in successful Stop trials with partial muscle bursts compared to those without (*p* < 0.05, TFCE-corrected; Fig. 6). This effect was specific to the final time bin (31 ms to SSRT), occurring in close temporal proximity to inhibition of the anticipated response and again supporting a functional link between β- and muscle bursting activity.

### Post hoc temporal analysis

Building on the observed association between β-burst rate and partial muscle bursting during successful Stop trials, we conducted a post hoc temporal analysis to explore the relative timing of cortical β-bursts and muscle bursts. Custom MATLAB scripts (2024b, The MathWorks) were used to extract the time bin in which each partial muscle burst occurred, alongside the directly adjacent time bins. Trials containing multiple partial bursts, as well as those in which a partial burst occurred in the final time bin (31 ms to SSRT), were excluded from the analysis.

**Figure 7.**
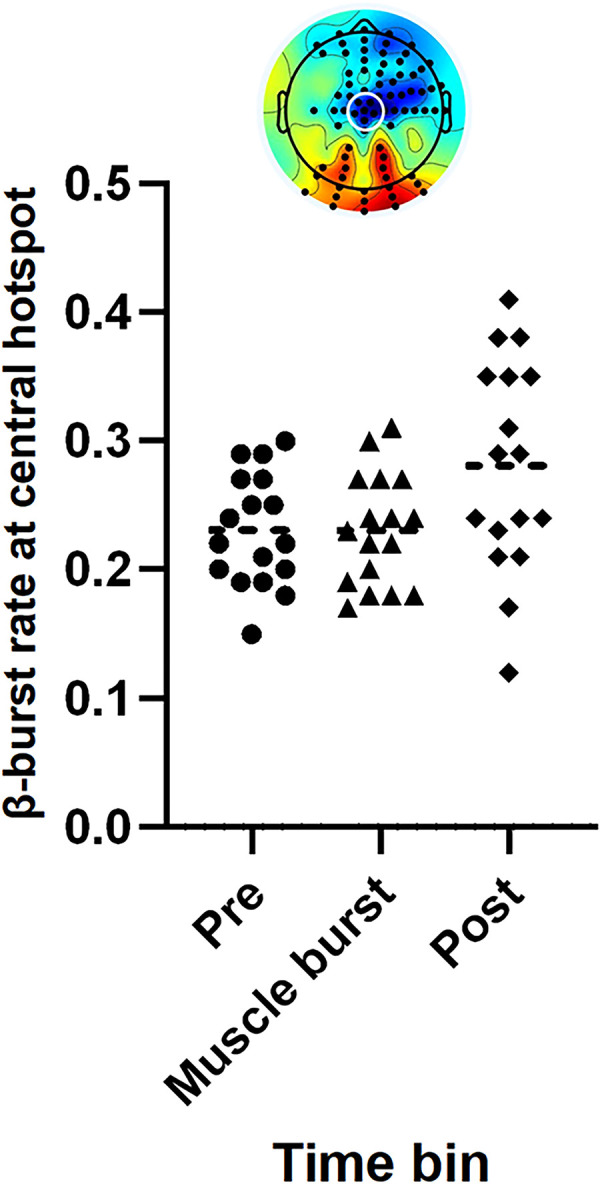
The mean β-burst rate over a central hotspot relative to muscle bursting. On successful Stop trials, a relative increase in β-bursting over central electrodes (Cz [A1] and surrounding electrodes) was apparent in the time bin following a partial burst, compared with before and during muscle bursting.

One-way ANOVAs were performed on β-burst rate values obtained in significant clusters identified in previous LMMs. There was no main effect of Time (*F*_(2,48)_ = 1.4; *p* = 0.249) on the β-burst rate over the significant cluster of right motor electrodes associated with partial muscle bursting in the final time bin (Fig. 6). There was a main effect of Time (*F*_(2,48)_ = 4.3; *p* = 0.019; *η*^2^ = 0.152) over the central β-bursting hotspot observed during response inhibition (Fig. 7), which demonstrated the significantly increased β-burst rate in the time bin following partial muscle bursts (0.28 ± 0.08), compared with the time bin in which the burst occurred (0.23 ± 0.04; *p* = 0.04) and the prior time bin (0.23 ± 0.04; *p* = 0.047). The β-burst rate was comparable in the time bins before and during muscle burst activity (*p* = 1.00).

## Discussion

The present study applied LMM–TFCE analysis to map β-burst activity during the ARIT and relate these neural events to muscle bursts in healthy older adults. Our approach elucidated distinct β-bursting patterns during response inhibition and withholding and uncovered a potential functional link between cortical β-bursting and late-stage gating of corticomotor drive. During response inhibition, our analyses revealed bilateral frontocentral β-bursting, demonstrating sensitivity to broad inhibitory network recruitment. Our brain-wide analysis further identified transient increases in parieto-occipital β-bursting, likely related to stop-signal processing before a functional shift from attentional to inhibitory networks. β-burst volume proved uniquely sensitive to mechanisms underlying response withholding, including left frontal activity, perhaps reflecting preparatory suppression to prevent premature movement. Collectively, we provide evidence that β-bursting is a temporally precise neural marker of inhibitory control processes in healthy aging. Combining β-burst detection with LMM–TFCE analysis provides a sensitive, trial-level framework to examine β-bursting measures in general and to explore inhibitory control in populations with heterogeneous responses.

### Response inhibition

Converging evidence supports a link between right frontal β-activity during response inhibition, as observed in the current study, and the right inferior frontal cortex (rIFC; Swann et al., 2009, 2012). Disruption of the rIFC around β-burst onset prolongs stopping latency (Hannah et al., 2020; Sundby et al., 2021), positioning the rIFC as a “brake” for motor output triggered by an unexpected event (e.g., the stop-signal; Aron et al., 2014). The rIFC, via the pre-SMA (Schaum et al., 2021), initiates corticosubcortical neural processes underlying inhibitory control via recruitment of antikinetic basal ganglia pathways (Rae et al., 2015; Diesburg et al., 2021). Specifically, reactive response inhibition is associated with rIFC-driven activation of the hyperdirect pathway (Jahanshahi et al., 2015; Chen et al., 2020), whereby excitation of the subthalamic nucleus from the pre-SMA increases inhibitory output from the globus pallidus internus (Coxon et al., 2012). The resultant net inhibition of thalamocortical circuitry enables rapid suppression of motor output (Jahanshahi et al., 2015). Communication through these frontobasal ganglia pathways occurs in the β-frequency band (Swann et al., 2009, 2012; Picazio et al., 2014; Wessel et al., 2016; Diesburg et al., 2021) with β-bursts emerging through temporally aligned convergence of synaptic input onto different cortical layers (Sherman et al., 2016; Bonaiuto et al., 2021), facilitating dynamic functional inhibition (Lundqvist et al., 2024). Our findings therefore reinforce the role of right frontal β-burst activity, driven by the rIFC and pre-SMA, as a neural index of inhibitory control.

While average β-power demonstrated stereotypical right frontocentral activity during response inhibition, β-bursting uncovered bilateral frontal activation. Often absent in response inhibition literature, select studies have linked the left IFC and pre-SMA to bimanual inhibition (Swick et al., 2008; Fiori et al., 2016; Schaffer et al., 2020). The robust analysis in the present study facilitated the identification of bilateral frontal contributions, implicating integrated contralateral and ipsilateral pathways involving the left IFC and pre-SMA in the inhibition of an internally generated bimanual response (Zhang et al., 2017). Comparison with a unimanual task version is required to confirm this interpretation. Bilateral frontal activity may also be interpreted as age-related compensatory mechanisms, where increased left IFC engagement may mitigate structural and/or functional changes in frontobasal ganglia circuitry discussed above (Coxon et al., 2012; Kang et al., 2021). Direct comparisons to younger cohorts will disentangle age-related changes in β-signatures. Nevertheless, these findings position β-bursting as a sensitive and mechanistically informative measure of inhibitory control, potentially revealing task- and age-dependent neural dynamics.

The observed temporal fluctuations in posterior β-bursting during response inhibition could be linked to attentional networks. An initial increase in the parieto-occipital β-burst rate was observed following the stop-signal. Parietal activity in motor control tasks links with attentive processing of task-relevant stimuli (Boehler et al., 2010), indicative of selective attending to the unexpected stop-signal. However, as the SSRT approached, β-burst activity over these areas was suppressed. This aligns with work by Enz et al. (2021), reporting a relative decrease in β-burst volume in centroparietal and occipital areas during successful Stop trials of the Stop-Signal task. The attenuation of posterior β-activity could minimize sensory or attentional “noise,” shifting toward optimized top–down inhibitory control.

In line with these temporally dynamic patterns, sensorimotor β-bursting followed partial muscle bursting on successful Stop trials, suggestive of a fast-acting mechanism in response to activation of the go process. We speculate that cortical inhibitory mechanisms, such as dynamic response threshold modulation by the pre-SMA (Wolpe et al., 2022), are exerted to rapidly suppress already-initiated motor activity. While increased cortical β-activity after muscle bursting could reflect general engagement of inhibitory control over time, our temporal analysis demonstrating comparable β-burst rates between the prior time bins suggests a functional link to the muscle burst itself. However, this analysis was exploratory. Trials with muscle bursts in the final time bin were omitted due to the lack of a subsequent comparison, and temporal variability was introduced, as muscle bursts occurred at varying points within each ∼31 ms bin. If sufficient trials with muscle bursting are obtained and the post-trial epoch is extended, future work could time-lock analyses to motor suppression, examining β-bursting both before and after CancelTime to capture the reactive mechanisms identified here. However, discarding successful Stop trials without motor activity risks omitting the most definitive examples of complete inhibition.

### Response withholding

The right frontocentral and sensorimotor β-burst rate was initially elevated during response withholding in Go trials, perhaps reflecting a tonically inhibited motor state, which is subsequently released to facilitate response initiation (Davranche et al., 2007; Sinclair and Hammond, 2008, 2009; Hannah et al., 2018). However, comparable response withholding is expected before stop-signal presentation in Stop trials. While time-locking β-bursts to individualized lift-times/SSRTs allowed direct comparison of response initiation and inhibition, this approach compromised temporal precision earlier in Stop trials, where the stop-signal was presented at variable times. It may therefore have been difficult to isolate the neural dynamics underlying response withholding during Stop trials.

β-burst volume appears more sensitive to response withholding mechanisms, revealing left frontal activity prior to the lift response. This activity may reflect preparatory suppression mechanisms from the IFC/pre-SMA, via the basal ganglia to intracortical M1 networks (Aron and Poldrack, 2006; Coxon et al., 2006) to prevent premature response initiation. Such mechanisms could not be discerned from average β-power or β-burst rate, and the activity was left-lateralized. Converging evidence suggests that the left hemisphere is specialized for predictive, feedforward control of movement dynamics (Sainburg, 2014). Within a “prepare-then-suppress” framework, proactive gating may therefore be stronger in left premotor–motor circuits.

The sensitivity of β-burst volume to response withholding processes was further evidenced through single-trial integration with muscle activity. The increase in parieto-occipital β-burst volume after premature muscle bursting (a “near-error”) may reflect feedback signaling from higher-order control regions to the visuoparietal cortex (Michalareas et al., 2016). β-bursting may signal transient functional inhibition (Engel and Fries, 2010; Lundqvist et al., 2024) for top–down stabilization of visuomotor mappings to prevent premature response release. Evidence indicates that β-bursts are morphologically heterogeneous, comprising distinct waveform motifs that may reflect different neural generators or computational roles (Rayson et al., 2023; Nougaret et al., 2024). Building on our novel findings mapping whole-brain β-bursting, burst detection methods that consider waveform shape may better isolate bursts from broadband activity (Seedat et al., 2020; Donoghue et al., 2022; Langford and Wilson, 2025) and provide further insight into the distinct β-mediated processes (Szul et al., 2023) underpinning the withholding and inhibition of responses.

### Clinical implications

The current findings support the use of LMM–TFCE analysis to characterize β-bursting patterns in populations with heterogeneous neural responses. This approach could enhance β-bursting analyses across a range of research contexts, including neural development (Rayson et al., 2023). More specifically, our β- (and muscle) bursting measures have potential as trial-by-trial markers of inhibitory function for characterizing impulse control issues in clinical populations. Individuals with Parkinson's disease exhibit elevated rates of premature and partial muscle bursts, alongside maladaptive modulation of corticomotor excitability, which is associated with impaired ARIT performance (Warden et al., 2025). These neurophysiological changes may reflect disrupted top–down β-burst signaling, compromising GABAergic M1 circuits, thereby failing to sufficiently suppress corticomotor excitability and resultant motor activity. Future research could investigate whether the observed β-burst patterns linked with response withholding and inhibition differ in clinical populations, evaluating their potential as markers of impulse control dysfunction.

## Conclusion

The current study highlights β-bursting as a temporally precise neural marker of inhibitory control in healthy older adults. Our novel use of a robust LMM–TFCE analysis framework identified distinct β-bursting patterns associated with both proactive response withholding and reactive inhibition, which were not captured by averaged β-power. These results underscore the sensitivity of the applied β-bursting analysis to both the timing and context of inhibitory demands, supporting its utility as a mechanistic and clinically relevant marker of inhibitory function. Future work with younger adults will discern which aspects reflect age-related changes versus universal inhibitory mechanisms detected by an advanced statistical approach.

## Data Availability

The anonymized datasets analyzed in the current study are available on the Open Science Framework: https://osf.io/4yu9n/.
